# A method to control phosphoinositides and to analyze PTEN function in living cells using voltage sensitive phosphatases

**DOI:** 10.3389/fphar.2015.00068

**Published:** 2015-03-31

**Authors:** Angeliki Mavrantoni, Veronika Thallmair, Michael G. Leitner, Daniela N. Schreiber, Dominik Oliver, Christian R. Halaszovich

**Affiliations:** Department of Neurophysiology, Institute of Physiology and Pathophysiology, Philipps-Universität MarburgMarburg, Germany

**Keywords:** phosphoinositides, signal transduction, cancer, fluorescence resonance energy transfer, voltage sensitive phosphatase (VSP), phosphatase and tensin homolog (PTEN), tumorigenic mutations, PI3-kinase signaling

## Abstract

Voltage sensitive phosphatases (VSPs), including engineered voltage sensitive PTEN, are excellent tools to rapidly and reversibly alter the phosphoinositide (PI) content of the plasma membrane *in vivo* and study the tumor suppressor PTEN. However, widespread adoption of these tools is hampered by the requirement for electrophysiological instrumentation to control the activity of VSPs. Additionally, monitoring and quantifying the PI changes in living cells requires sophisticated microscopy equipment and image analysis. Here we present methods that bypass these obstacles. First, we explore technically simple means for activation of VSPs via extracellularly applied agents or light. Secondly, we characterize methods to monitor PI(4,5)P_2_ and PI(3,4,5)P_3_ levels using fluorescence microscopy or photometry in conjunction with translocation or FRET based PI probes, respectively. We then demonstrate the application of these techniques by characterizing the effect of known PTEN mutations on its enzymatic activity, analyzing the effect of PTEN inhibitors, and detecting in real time rapid inhibition of protein kinase B following depletion of PI(3,4,5)P_3_. Thus, we established an approach that does not only allow for rapidly manipulating and monitoring PI(4,5)P_2_ and PI(3,4,5)P_3_ levels in a population of cells, but also facilitates the study of PTEN mutants and pharmacological targeting in mammalian cells.

## Introduction

Phosphoinositides (PIs) are membrane-bound signaling molecules essential for many cellular processes like migration, proliferation, membrane trafficking, and regulation of ion channels (Balla et al., 2009). Various experimental techniques have been developed to control PI levels in living cells, ranging from pharmacological approaches and genetic manipulations such as over-expression or knock-down of PI-metabolizing enzymes, to more dynamic approaches that allow temporal control over PI levels. Three distinct approaches for the acute manipulation of PI concentrations have been developed, all displaying certain limitations that need to be considered.

First, the FKBP-FRB heterodimerization system recruits a PI metabolizing enzyme (e.g., PI(4,5)P_2_-5-phosphatase) to the membrane when rapamycin is applied (Suh et al., 2006; Varnai et al., 2006). The timing of recruitment and consequently the change in, e.g., PI(4,5)P_2_ concentration can be controlled with precision on the order of seconds. However, recruitment and hence the alteration of PI levels is irreversible. Furthermore, the use of the rapamycin-induced dimerization system is limited to cell types that do not express large amounts of FKBP or FRB protein domains endogenously (Coutinho-Budd et al., 2013) and unwanted effects via endogenous targets of rapamycin have to be considered (Brown et al., 1994).

The second technique is similar, being based on the recruitment of PI-phosphatases to the membrane controlled by light-induced dimerization of CRY2 and CIBN domains (Idevall-Hagren et al., 2012). This approach allows for PI(4,5)P_2_ depletion that is reversible within minutes, but it has limitations concerning the analysis of PI levels using standard fluorescent biosensors, as the system is highly sensitive to blue and green light.

A third strategy to manipulate PI levels in living cells utilizes voltage sensitive phosphatases (VSPs) that have been isolated from several chordates (Murata et al., 2005; Hossain et al., 2008; Ratzan et al., 2011). VSPs consist of a voltage-sensing transmembrane domain (VSD) linked to a cytoplasmic phosphatase domain. Membrane depolarization activates the protein resulting in dephosphorylation of PIs. Although their biological function remains largely unknown, VSPs have proven to be highly useful to manipulate PI levels experimentally. For example, the *Ciona intestinalis* VSP (Ci-VSP), which primarily acts as a 5-phosphatase toward PI(4,5)P_2_and PI(3,4,5)P_3_ (Iwasaki et al., 2008; Halaszovich et al., 2009), has been used to study regulation of ion channels by PI(4,5)P_2_ (Lindner et al., 2011; Yudin et al., 2011).

The potency and versatility of this strategy has recently been expanded by the development of engineered voltage sensitive enzymes (Lacroix et al., 2011; Halaszovich et al., 2012). One such enzyme is the chimeric VSP PTEN_CiV_(termed “Ci-VSPTEN16” in (Lacroix et al., 2011)), where the PI(3,4)P_2_/PI(3,4,5)P_3_ 3-phosphatase PTEN (phosphatase and tensin homolog deleted from chromosome 10) replaces the phosphatase domain of Ci-VSP. PTEN_CiV_ is activated by depolarization of the membrane potential while fully retaining enzymatic properties of wild-type PTEN including substrate specificity (Lacroix et al., 2011). Accordingly, PTEN_CiV_ can be used as a tool to manipulate PI(3,4)P_2_ and PI(3,4,5)P_3_ levels.

Of note, PTEN itself has been the focus of extensive research efforts, since it is one of the most frequently mutated tumor suppressor genes in human cancer (Chalhoub and Baker, 2009) and in hamartoma-related syndromes (Hollander et al., 2011); PTEN dysfunction is also linked to autism spectrum disorders (ASDs) (Varga et al., 2009). Despite the significance of PTEN mutations in pathological conditions, there are only limited techniques to evaluate the effects of these mutations on the phosphatase activity of the protein. These include *in vitro* phosphatase assays and the functional analysis of PTEN in a yeast survival model (Cid et al., 2008). There is a need for methods to fully characterize PTEN activity in living mammalian cells. When combined with established fluorescence-based methods for reading out PI concentrations, the voltage-controlled PTEN_CiV_ chimera may provide an ideal platform for analyzing PTEN and its mutants in living cells.

One major drawback in using VSPs for analyzing PI functions and PTEN properties in living cells is the need for rather sophisticated methods and expertise for the control of the membrane potential, usually by patch clamp electrophysiology (e.g., Halaszovich et al., 2009; Sakata et al., 2011). Other disadvantages are the time consuming single cell technique and perturbation of the cell interior by whole-cell patch clamping. Here, we report the development of a novel experimental strategy to activate VSPs and PTEN_CiV_ without the use of electrophysiological instrumentation, enabling rapid, reversible, scalable, and broadly applicable manipulation of PI levels in a population of intact cells. Thus, we show that chemical or light induced activation of cation channels can be employed to depolarize the membrane potential and thereby activate Ci-VSP or PTEN_CiV_. Similarly, we present PTEN_CiV_ as a novel approach to study PTEN mutations and inhibitors with high precision. Furthermore, we demonstrate that PTEN_CiV_ can also be used to study the ubiquitous PI(3,4,5)P_3_/Protein kinase B (PKB, Akt) pathway on a time scale not easily accessible to biochemical approaches.

## Materials and methods

### Molecular biology

The chimeric construct PTEN_CiV_ was described previously (Lacroix et al., 2011). The Frubby construct was created from the Pippi-PI(4,5)P_2_ sensor kindly provided from Dr. M. Matsuda, Japan (Yoshizaki et al., 2007), by replacing the PLCδ_1_-PH domain with the tubbyC domain (Santagata et al., 2001). To remove PLCδ_1_-PH from the original plasmid we used the *BspE*1 and *Kpn*1 restriction sites. The same restriction sites were utilized to insert tubbyC into the construct. The amino acid sequence of this novel construct is provided as a Supplementary file. The AktAR construct was kindly provided from Dr. J. Zhang, Baltimore, USA (Gao and Zhang, 2008). Mutagenesis was performed using the QuickChangeII XL Site-Directed mutagenesis kit (Agilent Technologies, Santa Clara, CA).

### Plasmids and transfection

CHO and HEK 293 cells were plated on glass coverslips or—for TIRF experiments—glass bottom dishes (WillCo Wells B. V., Amsterdam, The Netherlands). For HEK 293 cells the glass was coated with poly-D-lysine. Cells were transfected 24–48 h later using JetPEI (Polyplus Transfection, Illkirch, France) as described (Halaszovich et al., 2009). For the used expression vectors see Supplementary Table S1. Bovine phosphatidylinositol 3-kinase p110α (constitutively active mutant K227E) was cotransfected with PTEN_CiV_ to increase PI(3,4,5)P_3_ levels except for experiments involving AktAR (Rodriguez-Viciana et al., 1996). In the latter experiments, PI(3,4,5)P_3_ levels were elevated by incubating the cells with 0.4 ng/μl insulin-like growth factor-1 (Sigma, St.Louis, MO) for 15 min (Halaszovich et al., 2009). HEK cells were preferred for confocal imaging, since they present a stronger membrane localization of PI markers. Experiments were performed at room temperature 24–48 h after transfection.

### Fluorescence microscopy

Confocal images were acquired using a Zeiss LSM710 Examiner upright microscope and a W-Plan-Apochromat 20 × 1.0 DIC M27 objective (Carl Zeiss AG; Jena; Germany). For excitation, laser lines of 561 nm for RFP and 488 nm for GFP were used. For detection, wavelength ranges were 582–754 nm for RFP and 493–582 nm for GFP. ChR2 was activated using an HBO lamp with excitation band-pass filters 470/40 nm (blue) and 436/20 nm (violet).

TIRF (Total Internal Reflection Fluorescence) imaging was performed by using a B×51WI upright microscope with a TIRF-condenser (Olympus, Hamburg, Germany) and a 488 nm laser (Picarro, Sunnyvale, CA). The setup was equipped with a LUMPIan FI/IR 40x/0.8- numerical aperture water immersion objective (Olympus), an IMAGO-QE cooled CCD camera (TILL Photonics GmbH, Grafelfing, Germany) and a monochromator (Polychrome IV, TILL Photonics).

Widefield illumination FRET (Förster Resonance Energy Transfer) was gained using a Nikon Eclipse TE2000-U inverted microscope equipped with a 40 × 0.55 LWD objective and coupled with an Oligochrome (TILL Photonics) light source. CFP and YFP were excited using a 430/24 nm (F49-430, AHF Analysentechnik, Tubingen, Germany) filter or a 500/20 nm (F49-500, AHF) filter respectively. Emission signal passed first through a tripleband beamsplitter CFP/YFP/mCherry (F68-017, AHF) and next detection area was selected using a viewfinder and a uEye camera (TILL Photonics). For CFP and YFP/FRET emission detection, a 470/23 nm (F49, AHF) and a 535/30 nm filter respectively, were placed before the photodiodes (TILL Photonics). Photocurrent was amplified by Oligochrome Imaging Control Unit, transmitted to an EPC-10 amplifier (HEKA electronics, Lambrecht, Germany) and data acquisition and analysis were performed with PatchMaster software (HEKA).

### Patch clamping

Whole-cell patch clamping was performed in CHO cells using an EPC-10 amplifier and PatchMaster (both HEKA). Patch pipettes' resistance was typically 1.5–4.0 MΩ, recordings were sampled at 20 kHz and low-pass-filtered at 5 kHz. For current clamp recordings, cells were held at 0 pA and changes of membrane voltage were recorded upon 1 s blue light exposure. PTEN_CiV_ was activated by 30 s voltage steps from a holding potential of −60 to +80 mV. Sensing currents from PTEN_CiV_ were measured as a result of a voltage jump to +120 mV from a holding potential of −70 mV with subtraction of linear leak and capacitive transient currents.

### Solutions and chemicals

In most experiments, cells were kept in extracellular solution containing (mM) 144 NaCl, 5.8 KCl, 0.9 MgCl_2_, 1.3 CaCl_2_, 0.7 NaH_2_PO_4_, 5.6 D-glucose, and 10 HEPES, pH 7.4 (with NaOH), 305–310 mosm/kg. Solutions with increased K^+^ concentration were made by replacing NaCl with KCl. For maximal depolarization, a 150 mM K^+^ solution was required prepared as follows (mM): 150 KCl, 0.9 MgCl_2_, 1.3 CaCl_2_, 0.7 NaH_2_PO_4_, 5.6 D-glucose, and 10 HEPES, pH 7.4 (with KOH). For the TRPV1 and ChR2(H134R) experiments Ca^2+^ was omitted from the standard extracellular solution to prevent the Ca^2+^ dependent activation of phospholipases-C (PLC) (Ca^2+^-free extracellular solution) (Rebecchi and Pentyala, 2000). Intracellular solution used for patch clamp experiments contained (mM) 135 KCl, 2.41 CaCl_2_, 2.5 MgCl_2_, 5 EGTA, 5 HEPES, and 3 Na_2_-ATP, pH 7.3 (with KOH), 290–295 mosm/kg.

Capsaicin and insulin-like growth factor-1 were purchased from Sigma-Aldrich, the bisperoxovanadate from Santa Cruz Biotechnology (Dallas, TX), DTT from Fermentas (St. Leon-Rot, Germany).

### Data analysis and statistics

**Figure 1:**
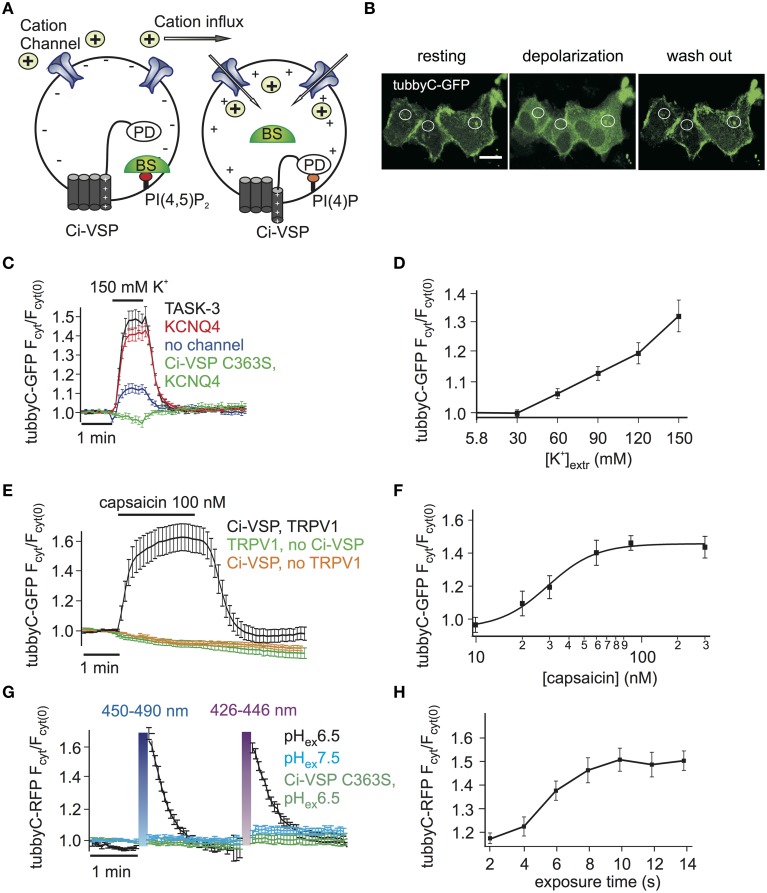
**Activation of Ci-VSP by membrane depolarization through overexpressed cation channels. (A)** Scheme representing the general strategy used to activate Ci-VSP. Cells co-express Ci-VSP, a PI biosensor and a cation channel. Cation influx into the cell results in membrane depolarization, activation of Ci-VSP, depletion of PI(4,5)P_2_ and lastly translocation of the sensor to the cytoplasm. BS, biosensor; PD, phosphatase domain. **(B)** Confocal images of HEK cells co-expressing Ci-VSP, KCNQ4 and tubbyC acquired before, during and after application of 150 mM K^+^. Fluorescence signal was recorded from cytoplasmic regions. Scale bar 20 μm. **(C)** Time series of relative increase of cytoplasmic fluorescence calculated from CHO cells expressing Ci-VSP, tubbyC-GFP and the K^+^ permeable channels TASK-3 (*n* = 48, *e* = 16), KCNQ4 (*n* = 82, *e* = 16) or no channel (*n* = 28, *e* = 9). The catalytically dead mutant Ci-VSP C363S (*n* = 23, *e* = 8) was used as negative control. tubbyC translocates to the cytoplasm upon 150 mM K^+^ application for 1 min. **(D)** K^+^ concentration-response curve from HEK cells co-expressing Ci-VSP, tubbyC-GFP, and KCNQ4 (*n* = 15, *e* = 6). **(E)** Application of 100 nM capsaicin in HEK cells co-expressing TRPV1 channel, Ci-VSP and tubbyC-GFP resulted in Ci-VSP activation and tubbyC translocation (*n* = 25, *e* = 8). No effect was observed in the absence of Ci-VSP (*n* = 19, *e* = 7) or TRPV1 (*n* = 16, *e* = 6). **(F)** Capsaicin concentration-response curve (*EC*_50_ = 29 ± 3 nM; at least 10 cells per concentration). **(G)** Activation of ChR2 with violet (426–446 nm) or blue (450–490 nm) light exposure for 10 s in HEK cells co-expressing tubbyC-RFP and Ci-VSP. tubbyC translocation was observed only at extracellular pH 6.5 (*n* = 12, *e* = 4), but not at pH 7.5 (*n* = 11, *e* = 4) or with the catalytically dead Ci-VSP C363S mutant (*n* = 23, *e* = 4). **(H)** Exposure time-response curve showing that 10 s of exposure with blue light resulted in maximal tubbyC translocation.

Confocal microscopy data acquisition was performed using ZEN2009 (Zeiss) software. For time series recordings, the fluorescence was averaged for regions in the cytosol (*F_cyt_*) and normalized to the baseline fluorescence prior to stimulation (*F*_0_). TIRF image analysis was performed using TILLvision software, and the background-corrected TIRF signal was normalized to *F*_0_. For FRET recordings, fluorescence intensity from a wide area including several cells—except for AktAR experiments, were fluorescence was collected from a single cell—was acquired and processed with PatchMaster. FRET ratios, calculated as fFRET/fCFP, were normalized to the baseline before stimulation. fCFP and fFRET were the signals from the CFP and FRET emission channels, corrected for the dark current recorded without illumination. IgorPro (Wavemetrics, Lake Oswego, OR) software was used for data analysis and presentation. To calculate the maximal response to K^+^ or capsaicin, values acquired during the last 30 s of the application were averaged. For ChR2-mediated Ci-VSP activation, the maximal response represents the first value acquired after the blue light exposure. To acquire the initiation kinetics using KCNQ4 and TRPV1, we calculated the time required to reach 50% of the maximal response [*t*_(1/2)_ of initiation], for each individual cell. For recovery kinetics, the time required to reach 50% of the baseline after the end of the application was calculated [*t*_(1/2)_ of recovery]. For ChR2 initiation kinetics analysis, we utilized the blue light exposure time curve (Figure 1H) to calculate *t*_(1/2)_ of initiation. Only cells that demonstrated a fluorescence intensity change greater than 4% upon application of the substance were used for the analysis. Error bars show the S.E.M, *n* represents the number of cells and *e* represents the number of experiments. For statistical analysis, a two-tailed Dunnett's test was performed to compare the single control to different experimental groups, two-tailed Student's *t*-test was used to compare two groups and Scheffe's test for multiple comparisons. Statistical significance was assigned at *P* < 0.05 and is marked as ^*^ for the Dunnett's and Scheffe's test and as # for Student's *t*-test.

## Results

### Activation of Ci-VSP in intact cells by membrane depolarization via cation channels

Activation of VSPs requires a depolarization of the membrane potential. To facilitate cell-based assays using VSPs we aimed at eliminating complex electrophysiological instrumentation such as patch clamp or two-electrode voltage clamp amplifiers from the experimental setting. To this end, we developed the experimental strategy depicted in Figure 1A, where cation channels are used to depolarize the cell membrane, thereby activating the VSP. The activity of the VSP can be monitored using fluorescence-tagged biosensor specific for the VSP's substrate (Varnai and Balla, 2006).

We started by exploring cation channel based strategies to activate the prototypical Ci-VSP, a PI(4,5)P_2_ 5-phosphatase. First, the K^+^ channels TASK-3 or KCNQ4 (Kv7.4) were used to depolarize the cells. These channels are expected to be largely or partially open under basal conditions, since TASK-3 is a constitutively open “leak” channel (Enyedi and Czirjak, 2010), whereas KCNQ4 is a voltage gated channel open at potentials above −50 mV, when expressed in CHO cells (Chambard and Ashmore, 2005). Given that both channels provide a substantial K^+^ conductance, cell depolarization can be simply achieved by increasing the extracellular K^+^ concentration.

Indeed, we observed PI(4,5)P_2_ depletion as reported by translocation of the PI(4,5)P_2_ biosensor tubbyC-GFP (Santagata et al., 2001) when 150 mM K^+^ was applied to cells co-expressing Ci-VSP and either KCNQ4 or TASK-3 (Figures 1B,C). Strong and rapid activation of Ci-VSP was achieved with either channel. Slightly more cells co-transfected with KCNQ4 (92%) than cells co-transfected with TASK-3 (80%) responded to application of K^+^. Therefore KCNQ4 was used for further experiments. When co-expression of channels was omitted, only 50% of the cells responded to application of K^+^ and changes in PI(4,5)P_2_ concentration were weak (Figure 1C), indicating that in CHO cells endogenous K^+^ channels were not sufficient to strongly drive Ci-VSP activity. The translocation of tubbyC-GFP was completely absent when catalytically dead Ci-VSP C363S was used (Figure 1C), confirming that K^+^-induced changes of PI(4,5)P_2_ were indeed mediated by activation of Ci-VSP.

VSPs can be used to deplete PIs in a gradual fashion by imposing graded membrane depolarization (Halaszovich et al., 2009). Membrane potential has a roughly logarithmic dependence on extracellular [K^+^] when the K^+^ conductance is dominant, suggesting that membrane potential and consequently Ci-VSP activity could be gradually increased by elevating extracellular [K^+^]. Indeed, when various K^+^ concentrations were washed in, Ci-VSP activity measured as the degree of translocation of the biosensor was also graded (Figure 1D). Maximum activity occurred at 150 mM K^+^, which was the maximum concentration we could achieve while keeping physiological osmolarity.

As an alternative approach to achieve membrane depolarization we utilized the capsaicin-activated TRPV1 channel (Figure 1E), a non-selective cation channel that depolarizes the membrane when active (Caterina et al., 1997). Because TRPV1 is also Ca^2+^ permeable and Ca^2+^ influx could potentially activate PLC (Rebecchi and Pentyala, 2000) causing PI(4,5)P_2_ cleavage, we used Ca^2+^-free extracellular solutions in these experiments. Application of capsaicin to HEK cells co-expressing TRPV1, Ci-VSP and tubbyC-GFP resulted in readily detectable PI(4,5)P_2_ depletion as indicated by tubbyC-GFP translocation, whereas no effect was observed in the absence of TRPV1 or Ci-VSP (Figure 1E). 100 nM of capsaicin was sufficient to induce the full response, as demonstrated by the capsaicin concentration-response curve (Figure 1F).

Over the last years, light-activated prokaryotic ion channels have been established as optogenetic tools for manipulation of the membrane potential (Lin, 2011). Among these channels, Channelrhodopsin-2 (ChR2), has been studied extensively and used widely to depolarize cells. ChR2 is a non-selective cation channel activated by visible light with a response spectral peak at around 450 nm (Nagel et al., 2003; Lin et al., 2009). Since activation by light could enable enhanced temporal control and may facilitate experimentation, we also attempted to use ChR2 to activate VSPs,

We first explored conditions for sufficient depolarization by ChR2. Extracellular acidification (pH 6.5) turned out to strongly increase the depolarization of cells expressing ChR2 when stimulated with blue light (Supplementary Figure S1), most likely due to the high H^+^ permeability of ChR2 (Nagel et al., 2003). This finding suggested low extracellular pH as the optimal condition to activate Ci-VSP via ChR2. Indeed, Ci-VSP was successfully activated only when extracellular solution of pH 6.5 was used but not at the standard extracellular pH 7.4 (Figure 1G). As in the case of TRPV1, we used Ca^2+^-free solution to prevent activation of PLC by Ca^2+^ influx. 10 s of blue or violet light exposure were sufficient for maximal stimulation of Ci-VSP, measured via translocation of tubbyC-RFP to the cytoplasm (Figure 1H). An RFP-tagged sensor was used in these experiments, because GFP excitation interferes with ChR2 activation.

**Figure 2:**
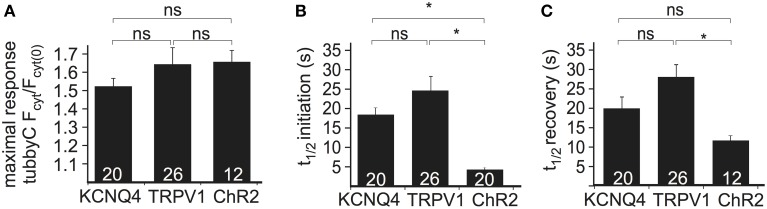
**Comparison of the three depolarization methods in HEK cells cotransfected with Ci-VSP, tubbyC, and one of the different channels. (A)** Bars representing the maximal response observed by overexpression of KCNQ4, TRPV1, and ChR2. For KCNQ4 we used the tubbyC translocation value from experiments as in graph Figure 1C, for TRPV1 from graph Figure 1E, and for ChR2 the maximal value from the blue light activation in graph Figure 1G. **(B)** Bars representing *t*_(1/2)_ of initiation, meaning the time required to reach 50% of the maximal response, for the three channels, calculated from data summarized in Figures 1C,E,H, respectively. **(C)** Bars showing *t*_(1/2)_ of recovery, the time required for 50% recovery to baseline. Numbers in columns represent the number of cells. ^*^ Indicates statistically significant difference; ns, not significant.

To compare the performance of the above methods of Ci-VSP activation, we evaluated the maximal response to each stimulus as well as the time required for PI(4,5)P_2_ depletion (i.e., sensor translocation) and the time required for PI(4,5)P_2_ recovery after cessation of the stimulus. Activation of all three channel types resulted in robust sensor translocation. Specifically, Ci-VSP activation induced by TRPV1 and ChR2 was slightly stronger (sensor translocation 1.63 ± 0.09- and 1.65 ± 0.06-fold, respectively), compared to the KCNQ4-mediated response (1.51 ± 0.04-fold increase; Figure 2A). However, these differences did not reach statistical significance. The fastest response (Figure 2B) was observed using ChR2 [*t*_(1/2)_ = 4.4 ± 0.4 s], followed by KCNQ4 [*t*_(1/2)_ = 18.2 ± 1.8 s] and TRPV1 [*t*_(1/2)_ = 25.0 ± 3.7 s]. For the recovery after stimulation, *t*_(1/2)_ was 12.4 ± 1.2 s for ChR2, 19.9 ± 2.7 s for KCNQ4 and 27.9 ± 3.1 s for TRPV1 (Figure 2C).

### Monitoring Ci-VSP activity without imaging

**Figure 3:**
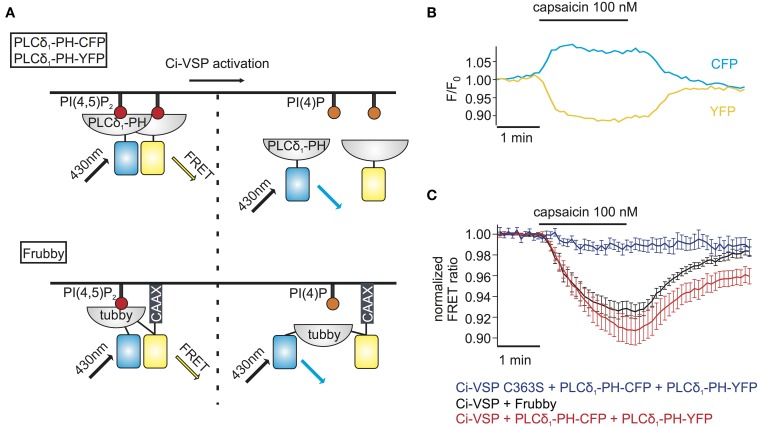
**FRET sensors to monitor Ci-VSP activation. (A)** Schematic representation of two different FRET sensors used to monitor Ci-VSP activity using photometry. PLCδ_1_-PH-CFP and PLCδ_1_-PH-YFP are located at the membrane before Ci-VSP activation and act as FRET donor and acceptor, respectively. FRET is disturbed when the sensors translocate to the cytoplasm after activation of Ci-VSP. Frubby is a construct that allows for intramolecular FRET and as shown in the schema it is held at the plasma membrane via a CaaX motif. **(B)** Representative trace of fluorescence signal from a cell expressing Ci-VSP, TRPV1 channel, PLCδ_1_-PH-CFP, and PLCδ_1_-PH-YFP. Treatment with capsaicin results in an increase of CFP and a decrease of FRET signal. **(C)** FRET ratio traces of the two FRET sensor systems. Both the PLCδ_1_-PH–CFP and PLCδ_1_-PH–YFP system (*e* = 15) and Frubby (*e* = 13) show a robust decrease of FRET ratio. No decrease in FRET is observed when the catalytically dead mutant Ci-VSP C363S (*e* = 6) is used.

The above method for monitoring PI depletion is based on the translocation of PI sensors between cytosol and plasma membrane. Detection of translocation requires imaging-based experiments, usually involving confocal microscopy or advanced microscopy techniques such as TIRF microscopy (van der Wal et al., 2001; Varnai and Balla, 2008; Halaszovich et al., 2009). Such approaches may not be optimal in many experimental settings including high-throughput assays, because experiments and analysis are based on single-cell data and thus are time-consuming, may be difficult to automatize and involve costly and complex equipment (e.g., confocal microscope, laser, TIRF-lenses). One way to circumvent these disadvantages is to employ PI sensors based on Förster resonance energy transfer (FRET): previously, pleckstrin homology (PH) domains of phospholipase C δ_1_ (PLCδ_1_-PH) fused to CFP and YFP have been used to detect PI(4,5)P_2_ concentration dynamics; dissociation of the domains into the cytosol increases their distance, thus reducing FRET efficiency (Figure 3A) (Varnai and Balla, 1998; Falkenburger et al., 2010; Hertel et al., 2011). As shown in Figures 3B,C, activation of Ci-VSP with capsaicin in cells co-expressing TRPV1, PLCδ_1_-PH-CFP, and PLCδ_1_-PH-YFP induced an increase of the CFP signal and a decrease of the FRET signal, indicating the decrease in FRET efficiency. Here, we used photodiodes to measure both CFP and YFP fluorescence emission, demonstrating that the FRET-based approach eliminates the need for microscopic spatial separation of membrane-bound and cytosolic fluorescence. We further sought to improve this approach, because consistency and reliability of this method may suffer from variable expression stoichiometry of both PH domains and from changes in fluorescence intensity resulting from sensor translocation. We therefore developed an intramolecular FRET sensor where binding of a PI(4,5)P_2_ binding domain to the membrane affects FRET efficiency between CFP and YFP flanking this domain (Figure 3A). Such a sensor based on PLCδ_1_-PH and anchored to the plasma membrane via a CaaX-box has been developed previously and termed Pippi-PI(4,5)P_2_ (Yoshizaki et al., 2007). However, dual sensitivity of the PLCδ_1_-PH sensor domain to both PI(4,5)P_2_ and IP_3_ may be a problem under some experimental conditions (Varnai and Balla, 2006). In contrast, the tubbyC domain used in the present study combines insensitivity to IP_3_ (Szentpetery et al., 2009) with higher sensitivity to changes in PI(4,5)P_2_ levels than PLCδ_1_-PH, as shown previously (Halaszovich et al., 2009). We thus designed a PI(4,5)P_2_ sensor, which we termed “Frubby,” where the PLCδ_1_-PH domain of the original Pippi PI(4,5)P_2_ sensor (Yoshizaki et al., 2007) was replaced by tubbyC as the PI(4,5)P_2_-binding domain. Depletion of PI(4,5)P_2_ using Ci-VSP under voltage clamp conditions showed that Frubby yields a robust, rapid, and readily reversible change in FRET ratio (Supplementary Figure S2A) as well as a graded response across a useful dynamic range (Supplementary Figure S2B).

We went on to combine detection of PI(4,5)P_2_ changes using Frubby with activation of Ci-VSP by TRPV1. As shown in Figure 3C, application of capsaicin to cells co-expressing Frubby, Ci-VSP and TRPV1 induced a strong FRET ratio change. Notably, signal amplitude was similar, but signal variability was smaller and reversibility was more complete compared to the bi-molecular FRET sensor (Figure 3C).

It should be noted that experiments using Ci-VSP to suppress PI(4,5)P_2_ dependent activity of ion channels such as GIRK and KCNQ2/KCNQ3 have demonstrated that the apparent PI(4,5)P_2_ affinity of these effectors lies within the dynamic range we demonstrated for Frubby (Supplementary Figure S2B) and the degree of PI(4,5)P_2_ depletion can be expected to be effective on those targets of PI(4,5)P_2_ signaling (cf. e.g., Murata et al., 2005; Iwasaki et al., 2008; Villalba-Galea et al., 2009; Rjasanow et al., submitted).

In conclusion, the combination of ligand-controlled activation of Ci-VSP together with FRET detection using the Frubby sensor allowed for the temporally precise depletion of PI(4,5)P_2_ in intact cells and concomitant monitoring of PI(4,5)P_2_ changes without the need for single-cell imaging.

### VSP-PTEN chimera for functional analysis of the tumor suppressor PTEN

PTEN is one of the most frequently mutated tumor suppressors in human cancer and exerts its tumor suppressor activity via direct antagonism of the phosphatidylinositol 3-kinase (PI3K) signaling pathway by counteracting the PI(3,4,5)P_3_ production via PI3K (Chalhoub and Baker, 2009; Varga et al., 2009; Hollander et al., 2011).

Development of the chimeric protein PTEN_CiV_, which fully reproduced the substrate specificity of wild-type PTEN and could be activated by membrane depolarization, provided a novel approach for controlling the activity of PTEN and studying this important tumor suppressor (Lacroix et al., 2011). Similarly to the approach we used for Ci-VSP, we attempted to activate PTEN_CiV_ without electrophysiological methods and compare the phosphatase activity of different PTEN mutants. We selected TRPV1 for activation of PTEN_CiV_ because high extracellular K^+^ concentrations interfered with some GFP-tagged biosensors (Supplementary Figure S3), precluding the use of KCNQ4 or TASK-3, and activation of ChR2 with blue light is incompatible with GFP-tagged sensors for PI(3,4,5)P_3_.

Since PTEN is a PI(3,4)P_2_/PI(3,4,5)P_3_ 3-phosphatase, we first tested various sensor domains that specifically bind to these 3-phosphorylated PIs for their suitability to quantitatively assess activity of PTEN_CiV_. Akt-PH-GFP (Servant et al., 2000) and Btk-PH-GFP (Salim et al., 1996) showed larger responses than GRP-PH-GFP (Klarlund et al., 1997) (Supplementary Figure S4). Because response kinetics were faster for Akt-PH than for Btk-PH (Supplementary Figure S4), we chose Akt-PH-GFP as the most suitable sensor for subsequent experiments.

**Figure 4:**
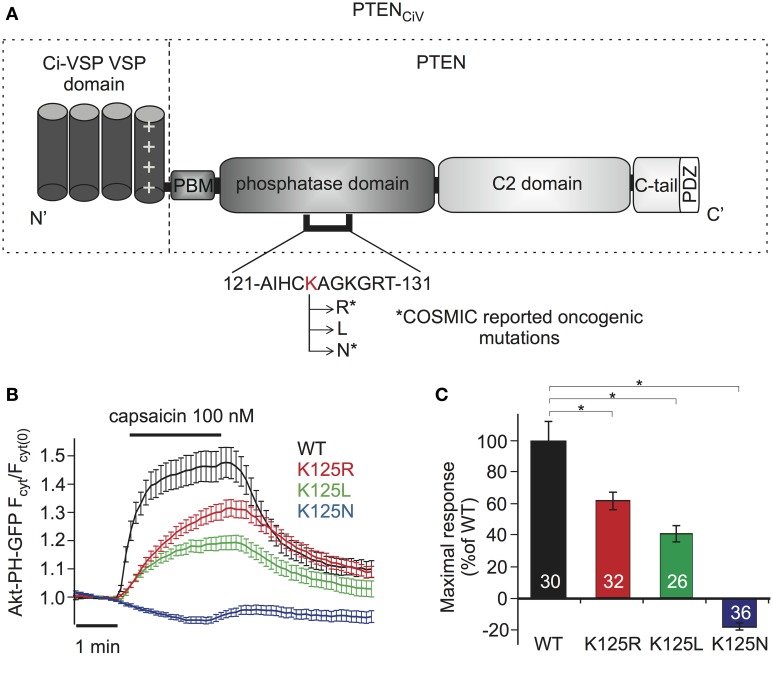
**Analyzing PTEN mutations using PTEN_CiV_ and TRPV1 mediated membrane depolarization. (A)** Schematic representation of PTEN_CiV_. The VSD of Ci-VSP, consisting of four transmembrane domains, was fused to the N terminal region of PTEN. Point mutations were introduced at K125 located in the P-loop of the catalytic domain. Asterisks mark oncogenic mutations as reported at COSMIC database (http://cancer.sanger.ac.uk/cancergenome/projects/cosmic/). PBM, PIP_2_ binding module; C-tail, the carboxyl-terminus domain; PDZ, postsynaptic density protein–*Drosophila* disc large tumor suppressor–zonula occludens 1 protein domain; P-loop, phosphatase binding loop. **(B**) Time series traces of Akt-PH-GFP translocation upon activation with capsaicin in cells co-expressing TRPV1 channel and WT or mutated PTEN_CiV_. **(C)** Mean of Akt-PH-GFP translocation calculated for the last 30 s of capsaicin application, normalized to the average response measured with WT PTEN_CiV_. Numbers in columns represent the number of cells. Statistically significant difference between WT and mutants is marked with *.

For examination of PTEN mutations by these methods, we focused on Lysine 125, located in the catalytic pocket of the protein (Figure 4A). PTEN mutations at this position are frequently found in cancer, according to COSMIC (Catalog of Somatic Mutations in Cancer, http://cancer.sanger.ac.uk/cancergenome/projects/cosmic/). In addition to oncogenic K125R and K125N, we also analyzed K125L because all three PTEN mutants were previously examined with a yeast survival model (Rodriguez-Escudero et al., 2011), thus allowing for direct comparison of methods.

When challenged by capsaicin-triggered depolarization, all three mutants showed impaired phosphatase activity compared to “wildtype” PTEN_CiV_ (Figure 4B). K125R and K125L presented significantly reduced enzymatic activity, yielding maximum sensor translocation of 61.17 ± 5.48% and 40.43 ± 5.11% relative to “wildtype” PTEN_CiV_ activity respectively, while the K125N mutation resulted in a complete loss of enzymatic function (Figure 4C).

### PTEN_CiV_ can be exploited to test PTEN inhibitors

Bisperoxovanadium (bpV) compounds are specific PTEN inhibitors and have been widely used to assess the role of PTEN in cellular processes (Schmid et al., 2004). They have been characterized using *in vitro* phosphatase assays and Western blot analyses of the phosphorylation of PKB (Protein Kinase B, Akt), a downstream target of the PTEN pathway. We considered these compounds as a useful example for exploring the potency of the PTEN_CiV_/live-cell approach in analyzing the effect of PTEN modulators in intact cells. We examined the effects of two compounds, bpV(HOpic) where bpV is attached to a polar functional group and bpV(phen) where bpV is attached to a neutral functional group.

TRPV1 was used to activate PTEN_CiV_ as described above. Here, we measured membrane association of the PTEN activity sensor Akt-PH-GFP by TIRF microscopy (Lacroix et al., 2011). TIRF selectively captures fluorescence from the cell membrane, thus dissociation of the PI sensor used to monitor PTEN_CiV_ activation results in a decrease of the fluorescent signal.

**Figure 5:**
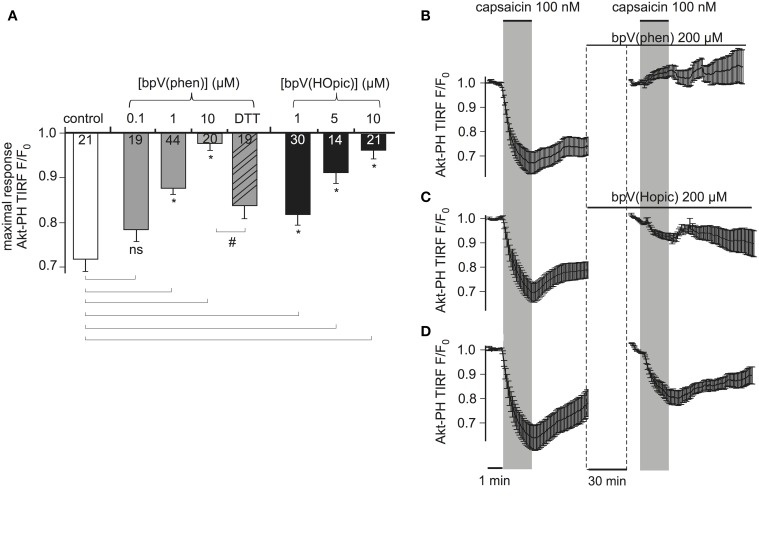
**PTEN_CiV_ is inhibited by bisperoxovanadate compounds. (A)** Bars representing the effect of overnight incubation of bisperoxovanadate compounds on PTEN_CiV_ activity. Fluorescence intensity was acquired with TIRF microscopy of HEK cells expressing PTEN_CiV_, TRPV1 channel, and Akt-PH-GFP. Decrease in the fluorescence signal recorded from the membrane is observed upon capsaicin application. Cells were treated with different concentrations of the PTEN inhibitors bpV(phen) or bpV(Hopic). Cells that after an overnight incubation with 10 μM bpV(phen) were washed out and incubated for 3 h with 5 mM of the reducing agent DTT showed a partial recovery of phosphatase activity. **(B–D)** Graph demonstrating the effect of the inhibitors in real time. Data is normalized to the time intervals immediately preceding each capsaicin application. See Supplementary Figure 5 for a presentation of the data without re-normalization for the second agonist application. **(B)** HEK cells that had initially responded to a 100 nM capsaicin activation were incubated for 30 min with 200 μM of the inhibitor bpV(phen). After the 30 min incubation they presented no response to a second capsaicin application (*n* = 11, *e* = 4). **(C)** Application of the inhibitor bpV(Hopic) also impairs PTEN_CiV_activity (*n* = 10, *e* = 4). **(D)** Graph showing a control experiment where no inhibitor was applied between the two capsaicin applications (*n* = 9, *e* = 3). Statistically significant difference between control and treated samples is marked with ^*^, while the # sign indicates significant difference between the 10 μM bpV(phen) and the DTT treated samples. Numbers in columns represent the number of cells.

First, cells were incubated with either bpV compound overnight and throughout the experiment. As shown in Figure 5A, both compounds inhibited PTEN activity in a dose-dependent manner. Nearly complete inhibition was observed with 10 μM of bpV(phen) or bpV(HOpic). Inhibition was partially reversible when cells that were incubated overnight with 10 μM of bpV(phen) were washed and incubated with 5 mM of the reducing agent Dithiothreitol (DTT) for 3 h.

To investigate the time course of the inhibition, which is less easily addressed by conventional biochemical methods, we monitored the same cells before and after application of inhibitors. Cells responsive to a first stimulation by capsaicin were incubated for 30 min with 200 μM bpV(phen) (lower concentrations failed to inhibit PTEN_CiV_ in acute application, data not shown). These cells did not respond to a second application of capsaicin indicating the inhibition of PTEN_CiV_ (Figure 5B, Supplementary Figure S5). Similarly, 30 min application of 200 μM of bpV(HOpic) greatly decreased the phosphatase activity (Figure 5C, Supplementary Figure S5). Control experiments were performed where no inhibitor was applied between subsequent capsaicin treatments (Figure 5D, Supplementary Figure S5). In that case, the second response—although decreased—remained pronounced. This decreased response might be explained by incomplete recovery of PI(3,4,5)P_3_ levels (Supplementary Figure S5, lower trace). The much lower efficacy of bpV in acute application when compared to overnight incubation may indicate that permeation into the cell membrane is slow. Indeed, when the inhibitor was applied intracellularly, the inhibitory concentrations were similar to the concentrations required with overnight incubation (Supplementary Figure S6).

### PTEN_CiV_ activation allows for rapid control of PI3K downstream pathways

**Figure 6:**
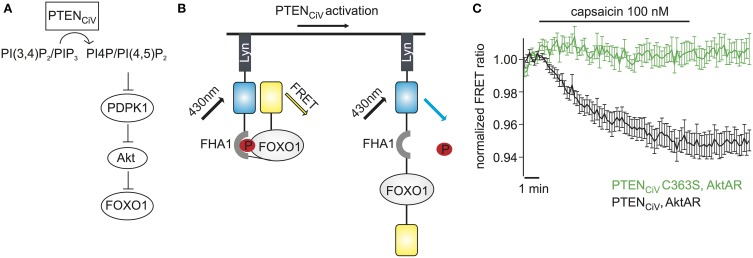
**Activation of PTEN_CiV_ leads to rapid dephosphorylation of FOXO1. (A)** PTEN_CiV_ activation decreases PI(3,4)P_2_/PI(3,4,5)P_3_ levels which results in Akt's displacement from the plasma membrane. This displacement prevents the phosphorylation and activation of Akt by PDPK1 (3 phosphoinositide dependent kinase 1). Akt is then unable to phosphorylate downstream targets like FOXO1. **(B)** Schematic representation of the Akt activation reporter, AktAR. FOXO1 at the phosphorylated state dimerizes with the Forkhead associated (FHA) domain and results in approach of the fluorophores. Activation of PTEN_CiV_ should disrupt this interaction. **(C)** FRET ratio traces of cells expressing TRPV1, PTEN_CiV_, and AktAR. Activation of PTEN_CiV_ with 100 nM of capsaicin for 10 min results in a decrease of the FRET signal, indicating the rapid dephosphorylation of FOXO1 (*n* = 11, *e* = 4 for PTEN_CiV_; *n* = 9, *e* = 3 for PTEN_CiV_ C363S).

The major role of PTEN is to antagonize the phosphatidylinositol 3-kinase (PI3K) signaling pathway. This pathway is controlled by the serine-threonine protein kinase Akt (also known as protein kinase B) which is recruited and activated by plasma membrane PI(3,4,5)P_3_ and in turn regulates a plethora of downstream targets through a cascade of protein kinases (Brazil et al., 2004) (Figure 6A).

We wondered if our method to rapidly change PTEN activity might have the potential to interrogate the dynamics of PI3K signaling. To assess the effect of PTEN_CiV_ on downstream targets of the Akt signaling pathway we utilized the Akt activity reporter, AktAR, described by Gao and Zhang (2008). AktAR is a membrane-anchored FRET sensor to monitor the kinase activity of Akt that is composed of part of the Akt-recognition sequence of FOXO1 and a forkhead-associated domain (FHA1). Akt-dependent phosphorylation of FOXO1 leads to dimerization of the two domains and an increase in the FRET signal, i.e. the FRET ratio directly correlates to the kinase activity of Akt (Figure 6B). In cells co-expressing TRPV1, PTEN_CiV_, and AktAR, application of capsaicin for 10 min indeed led to a decrease of the FRET signal indicating decreased Akt activity. In contrast, no decrease was observed when catalytically-dead PTEN_CiV_ C363S was co-expressed (Figure 6C). In conclusion, ligand-induced activation of PTEN_CiV_ does not only allow manipulation of PI(3,4,5)P_3_ at the plasma membrane, but also control and analysis of the downstream signaling cascade, including phosphorylation of Akt substrates in intact living cells.

## Discussion

We developed a fast, simple, reversible, and scalable method that can be used not only as a tool to control PI levels in living cells, but also to study the tumor suppressor PTEN. The approach is based on well characterized VSPs, namely the PI(4,5)P_2_ 5-phosphatase Ci-VSP (Iwasaki et al., 2008; Halaszovich et al., 2009) and the engineered PI(3,4)P_2_/PI(3,4,5)P_3_ 3-phosphatase PTEN_CiV_ (Lacroix et al., 2011). To activate these enzymes in living cells, the membrane potential must be depolarized. So far, this required the use of electrophysiological techniques, usually whole-cell patch clamping for mammalian cells. Since such methods are not available to many laboratories, either because of lack of equipment or electrophysiological expertise, we sought to find a simpler and broadly applicable method for membrane depolarization. Our data demonstrate that activation of overexpressed cation channels is a simple and powerful method to activate Ci-VSP or chimeric VSPs and thereby manipulate PI(4,5)P_2_ and PI(3,4,5)P_3_ concentrations in a temporally well-controlled manner.

Currently, widely used methods for acutely altering PI concentrations are based on the recruitment of PI metabolizing enzymes to the membrane through a dimerization strategy, triggered either by rapamycin (Suh et al., 2006; Varnai et al., 2006) or via an optogenetic approach (Idevall-Hagren et al., 2012). Compared to these methods, the main advantage of the VSP-based approach is the reversibility of PI changes with times for 50% recovery of 12 to 28 s. In contrast, the rapamycin system is virtually irreversible and with the optogenetic method half-maximal recovery of PI(4,5)P_2_, measured as either sensor domain translocation or KCNQ2/3 current inhibition, required several minutes (Idevall-Hagren et al., 2012). One additional disadvantage of the optogenetic approach is that its sensitivity to blue and green light places restrictions on which fluorophores can be used for imaging / monitoring purposes. On the other hand it allows for subcellular manipulation of PI concentrations, whereas VSP activation in our system always affects the entire plasma membrane of the cell. We further note that activation of VSPs by each of the tested ion channels allowed for a graded depletion of PI(4,5)P_2_. This high level of control over PI(4,5)P_2_ levels may be used to compare the apparent affinity of intracellular PI(4,5)P_2_ effectors in a semi-quantitative manner, as we have demonstrated in earlier work (Halaszovich et al., 2009).

Each of the three classes of channels explored here activated Ci-VSP with similar efficacy, which was assessed as degree of translocation of the PI(4,5)P_2_ sensor tubbyC, tagged with either GFP or RFP. Although not being a direct quantification of actual [PI(4,5)P_2_], this method provides a sufficient means of comparison of degree of PI(4,5)P_2_ depletion. While we cannot exclude the possibility that the choice of the tag, which was based on the technical requirements discussed below, influences the degree of fluorescence increase seen in the experiments, we expect the main determinant to be the properties of the PI(4,5)P_2_ binding domain itself. Therefore a meaningful comparison of the degree of Ci-VSP activity can be drawn from experiments even when differently tagged sensors are used.

Advantages and limitations of each channel type mostly relate to the technical aspects of channel activation. Depolarization through constitutively open K^+^ channels requires full exchange of bath solution to alter the K^+^ concentration; in some cases, high extracellular K^+^ concentrations interfered with GFP fluorescence (Supplementary Figure S3). Activation of ligand gated channels does not necessarily require complete bath exchange, since the ligand can be added to the bath solution, especially when small concentrations can be used; for the TRPV1 channel used here, an agonist concentration as low as 100 nM was sufficient for maximal effect. For plate-reader systems, there is equipment available to perform such an application of a ligand to the assay. A disadvantage of using TRPV1 is the requirement for a Ca^2+^-free bath solution, although in many cases this will not be a severe restriction. Also, use of TRPV1 was the method showing the slowest kinetics, which is probably related to ligand permeation and binding/unbinding. Manipulations of the bath solution can be avoided altogether when the optogenetic approach using a light gated channel (ChR2) is used. Here, we achieved the fastest response times. Disadvantages of using ChR2 are that a low pH (6.5) and Ca^2+^-free bath solution had to be used and that the spectral characteristics of ChR2 preclude the use of certain wavelengths for excitation of fluorophores. Notably, we were not able to use GFP-tagged PI-sensors and used RFP instead. We would like to point out that naturally we could only study a subset of ion channels that potentially can be useful for VSP activation. Further candidates like e.g., the FMRFamide-gated sodium channel FaNaC (Lingueglia et al., 1995), which has been used to depolarize tsA201 cells as well as to activate mammalian neurons (Scheel et al., 2005; Schanuel et al., 2008), might be better suited for specific applications and should be considered accordingly. One caveat when using ion channels for VSP activation is the PI(4,5)P_2_ dependence of some of these channels. Regarding those used in this study, KCNQ4 shows inhibition following PI(4,5)P_2_ depletion, in contrast to the PI(4,5)P_2_ insensitive TASK-3 (Lindner et al., 2011), also TRPV1 has been described as PI(4,5)P_2_ sensitive (Klein et al., 2008). While the PI(4,5)P_2_ sensitivity of these two channels was not sufficient to make them unsuitable for the purpose of VSP activation, for more sensitive channels or in cell systems showing lower baseline PI(4,5)P_2_ concentrations this might not be the case. One such example might be the highly sensitive R555C mutant of KCNQ1, where even basal Ci-VSP activity is reported to be sufficient for complete channel deactivation (Coyan et al., 2014). Therefore when deviating from the ion channels or cell systems used in the present study their suitability should be carefully assessed.

Beyond manipulation of PI(4,5)P_2_ and PI(3,4)P_2_/PI(3,4,5)P_3_ levels, the availability of PTEN_CiV_ considerably extends the range of useful applications of the method presented here. The role of PTEN in pathological conditions like tumors of sporadic and germline origin and ASDs is well established (Cantley and Neel, 1999; Varga et al., 2009; Hollander et al., 2011). Biochemical *in vitro* techniques or a heterologous yeast reconstitution system have usually been used to investigate the effect of mutations on protein function or to study chemical PTEN inhibitors and activators.

The method we present should provide a useful complementation of the current spectrum of methods. We note several potential advantages of this approach. First, our method proved to be more sensitive than the published yeast-based method (Rodriguez-Escudero et al., 2011) in assessing the effect of PTEN mutations (see below). Second, in the living mammalian cell, PTEN acts on its substrates in the native membrane environment, which can differ substantially from the composition of vesicles used to present the potential substrates or even soluble substrate analogs used in *in vitro* assays. This might affect activity or substrate specificity inferred from such experiments. For example, the human VSP ortholog hVSP1 (also known as TPIP or TPTE2) was first described as a PI(3,4,5)P_3_ 3-phosphatase based on *in vitro* data (Walker et al., 2001). However, recent experiments in mammalian cells using a voltage sensitive hVSP1 chimera revealed that it is a PI(4,5)P_2_/PI(3,4,5)P_3_ 5-phosphatase (Halaszovich et al., 2012). Finally, the new method is applicable to a wide range of cell types, including cell lines derived from human tumors, once heterologous expression of the molecular components (VSP and cation channel) can be achieved in that cell line.

For demonstration purposes, we studied PTEN mutations, including known oncogenic mutations. For some of these PTEN mutations, results obtained with the PTEN_CiV_ approach were consistent with previously published findings from *in vitro* and yeast assays (Rodriguez-Escudero et al., 2011). Thus, PI(3,4,5)P_3_ phosphatase activity was strongly decreased for PTEN_CiV_ K125L and abrogated for PTEN_CiV_ K125N (Figures 4B,C). However, the yeast survival method did not detect any defect of the PTEN K125R, although the mutant is annotated as an oncogenic mutation. Our approach indeed detected a decrease of phosphatase activity by 40%. This finding might explain the tumorigenic effect of that mutation and also highlights the sensitivity of our approach. As ASD-related mutations have been shown to decrease but not abrogate PTEN phosphatase activity (Rodriguez-Escudero et al., 2011), this high sensitivity should render our method a well-suited tool for testing ASD- and tumor-associated PTEN mutations.

Similarly, the PTEN_CiV_ method enabled analysis of pharmacological manipulation of PTEN. *In vitro* and *in vivo*, bpV compounds inhibit PTEN at nanomolar concentrations with minimal cytotoxicity (Schmid et al., 2004). In that study, more indirect methods like assessing the phosphorylation state of Akt/PKB were used, whereas we directly monitored PTEN's substrate PI(3,4,5)P_3_ to determine the effect of these inhibitors in living cells. The bpV compounds have been used to study the involvement of PTEN in signaling, proliferation, apoptosis, and neurogenesis (Band et al., 1997; Batty et al., 2007; Pi et al., 2012; Tian et al., 2012; Becker et al., 2013); however, higher concentrations (200 nM–100 μM) were required. We found comparable concentrations to effectively inhibit PTEN activity in our live cell assay. This technique holds promise for high-throughput screens for novel substances with pharmacological activity toward PTEN. Such substances could also include enhancers of PTEN activity, which might be used for anticancer therapy.

These findings demonstrate that PTEN_CiV_ can be used to study mutations and compounds affecting the catalytic domain of PTEN. While this technique has clear advantages in terms of simplicity and through-put over existing techniques, it might be unable to detect the effect of mutations or interactions outside the catalytic domain which might affect PTEN activity, e.g. by interfering with membrane association. Therefore we view the VSP-based method not as a complete replacement for, but as a complement to existing methods.

Moreover, because the PTEN_CiV_ method is essentially a real-time approach in living cells, it can yield insights into the temporal cellular dynamics of PI(4,5)P_2_ and PI(3,4,5)P_3_ signaling that are difficult to obtain with a purely biochemical approach. As an example, we showed that down-regulation of PI(3,4,5)P_3_ (and PI(3,4)P_2_) concentration resulted in a surprisingly rapid switching-off of signaling by dephosphorylation of intermediates within the Akt signaling cascade, which (to our knowledge) has not been appreciated before. This finding points to a rather high activity of the protein phosphatase(s) that antagonize Akt kinase activity.

In summary, we presented a series of simple to use and broadly applicable methods that provide temporal control of VSPs. This allows for manipulation of PI levels and subsequently manipulation of their downstream targets. Moreover, using the engineered voltage sensitive enzyme PTEN_CiV_ we suggest a novel method for charactering this critical tumor suppressor. Further development of a method combining PTEN_CiV_ with an optimized FRET sensor, could create a powerful automated tool to screen for pathological PTEN mutations and for substances affecting activity of PTEN.

## Grant support

Supported by University Medical Center Giessen and Marburg grant UKGM 32/2011 MR to CRH, grant UKGM 17/2013 to MGL, and Deutsche Forschungsgemeinschaft / DFG grant SFB593 TP A12 to DO.

### Conflict of interest statement

The authors declare that the research was conducted in the absence of any commercial or financial relationships that could be construed as a potential conflict of interest.

## Abbreviations

ASDs: autism spectrum disorders
bpV: bisperoxovanadium
C-tail: the carboxyl-terminus domain
ChR2: channelrhodopsin-2
Ci-VSP: Ciona intestinalis voltage sensitive phosphatase
COSMIC: Catalogue of Somatic Mutations in Cancer
FHA1: forkhead-associated domain
FOXO1: forkhead box 1
FRET: Förster resonance energy transfer
P-loop: phosphatase binding loop
PBM: PIP2 binding module
PDZ: postsynaptic density protein-Drosophila disc large tumor suppressor-zonula occludens 1 protein domain
PH: pleckstrin homology
PI: phosphoinositide
PI3K: phosphatidylinositol 3-kinase
PKB: protein kinase B
PTEN: phosphatase and tensin homolog deleted from chromosome 10
PTENCiV: PTEN with Ciona intestinalis voltage sensor domain
TIRF: total internal reflection fluorescence
VSD: voltage sensor domain
VSP: voltage sensitive phosphatase.
