# Supplementary material for: A method to control phosphoinositides and to analyze PTEN function in living cells using voltage sensitive phosphatases
Source: Front Pharmacol. 2015 Mar 31;6:68. doi: 10.3389/fphar.2015.00068 (PMC4379879; doi:10.3389/fphar.2015.00068)

Mavrantoni et al.: A method to control phosphoinositides and to analyze PTEN function in living cells using voltage sensitive phosphatases

Supplementary legends

Sup. Table S1. Constructs used in this study.

Sup. Fig. S1. ChR2-mediated membrane depolarization. Representative current clamp recording from a CHO cell expressing ChR2, without current injection (I_{hold} = 0 pA). Membrane depolarization increased under low pH extracellular conditions.

Sup. Fig. S2. Functional characterization of Frubby using patch clamping. Experiments were performed on CHO cells expressing Frubby and Ci-VSP. **A:** change in average FRET ratio, normalized to the baseline ratio, in response to membrane depolarization (n=7, e=7). **B:** change in normalized FRET ratio in response to graded membrane depolarization (n=11, e=11). The continuous curve represents a Boltzmann-function ($y = y_{min} + (y_{max} - y_{min})/(1 + \exp((V - V_{1/2})/s))$) fitted to the data. Membrane voltage for half maximal response $V_{1/2}$ = -3.4 ± 2.5 mV, as estimated from the fit. This value is comparable to the one we found for tubbyC-GFP translocation in previous experiments ($V_{1/2}$ = -4.6 ± 2.9 mV, cf. ref. (10)).

Sup. Fig. S3. Activation of $PTEN_{CiV}$ using K^+ dependent depolarization. **A**: Time series of cytoplasmic fluorescence of cells transfected with $PTEN_{CiV}$ and KCNQ4. Application of 150 mM K^+ causes a decrease in the GFP signal in control experiments with $PTEN_{CiV}$ C363S or in experiments performed with GRP1-PH-GFP, a domain with low membrane affinity (n=34, e=19 for Akt-PH; n=15, e=12 for Btk-PH; n=26, e=15 for $PTEN_{CiV}$ C363S; and n=14, e=8 for GRP1-PH). **B**: Control experiment where free GFP was co-expressed with $PTEN_{CiV}$ and KCNQ4. The cytoplasmic fluorescence signal also decreased upon K^+ application. This implies that the decreases seen in panel A is most likely not due to an actual sensor translocation (n=22, e=11). **C**: Confocal images of HEK cells expressing different PI sensors. The PIP_2 sensor tubbyC-GFP is strongly located to the membrane compared to $PI(3,4,5)P_3$ sensors Akt-PH-GFP, Btk-PH-GFP, and GRP1-PH-GFP. Since we recorded the fluorescence signal from the interior of the cell, it is possible that the fluorescence decrease observed in panels A and B would be more pronounced with those domains that show higher cytosolic fluorescence under basal conditions and vice-versa was not

apparent in previous experiments using tubbyC and PLCδ_1-PH (cf. fig. 1) that are strongly membrane localized under resting conditions. Scale bar 15 µm.

Sup. Fig. S4. Selection of an PI(3,4,5)P_3 marker for monitoring $PTEN_{CiV}$ activity. Cells expressing $PTEN_{CiV}$ and TRPV1 channel were used to compare the different PI(3,4,5)P_3 sensors Btk-PH-GFP (n=36, e=26), Akt-PH-GFP (n=31, e=16), and GRP1-PH-GFP (n=19, e=14). Akt-PH and Btk-PH presented the biggest responses. Akt-PH that recognizes PI(3,4)P_2 and PI(3,4,5)P_3 shows faster kinetics of activation and recovery. No effect was observed with the catalytically dead $PTEN_{CiV}$ C363S (n=14, e=7).

Sup. Fig. S5. $PTEN_{CiV}$ is inhibited by bisperoxovanadate compounds (data presented without re-normalization). Same data as presented in fig. 5 B-D but without re-normalization to the time interval immediately preceding the second capsaicin application.

Sup. Fig. S6. bpV(phen) and bpV(HOpic) abolish $PTEN_{CiV}$ activity at micromolar concentrations when applied intracellularly. **A**: Representative recordings from patch clamped CHO cells expressing $PTEN_{CiV}$ and Akt-PH-GFP. 10 µM of inhibitor in the pipette completely abolish $PTEN_{CiV}$ phosphatase activity when $PTEN_{CiV}$ was activated with a voltage jump from -60 mV to +80 mV for 30 seconds. **B**: Sensing current recorded at +120 mV from a holding potential of -70 mV from a cell where 10 µM of bpV (phen) was applied though the patching pipette. **C, D**: Dose response curve for bpV (phen) and bpV (Hopic) derived from experiments as in panel A.

Supplementary Table S1. Constructs used in this study

Name	UniProt accession No.	Aminoacids	Vector
Biosensors			
PLCδ1-PH	P51178	1-170	pEGFP-N, pEYFP-C, pECFP-C
Btk-PH	Q06187	1-177	pEGFP-N
Akt1-PH	P31749	1-164	pEGFP-N, pECFP-N, pEYFP-N
GRP1-PH	O43739_2	261-381	pEGFP-C
tubbyC	P50586_2	243-505	pEGFP-C, pRFP-C
Frubby	Chimeric construct		pCAGGS
AktAR	Chimeric construct		pcDNA3
Channels			
KCNQ4	P56696	Full length	pcDNA3.1
TASK-3	Q9NPC2	Full length	pcDNA3.1
TRPV1	Q35433	Full length	pEYFP-N (stop codon before YFP)
ChR2(H134R)	B4Y105	Full length	pEYFP
Enzymes			
Ci-VSP	Q4W8A1	Full length	pRFP-C, pECFP-C
$PTEN_{civ}$	Chimeric construct		pRFP-C
PI3-kinase p110α (K227E)	P32871	Full length	pcDNA3.1

Supplementary Figure S1. ChR2-mediated membrane depolarization

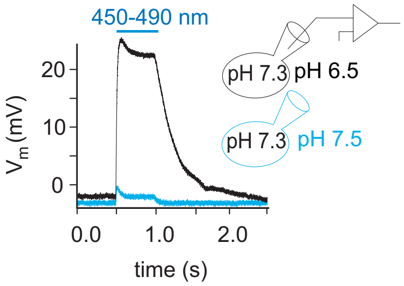

Supplementary Figure S2. Frubby functional characterization using patch clamping

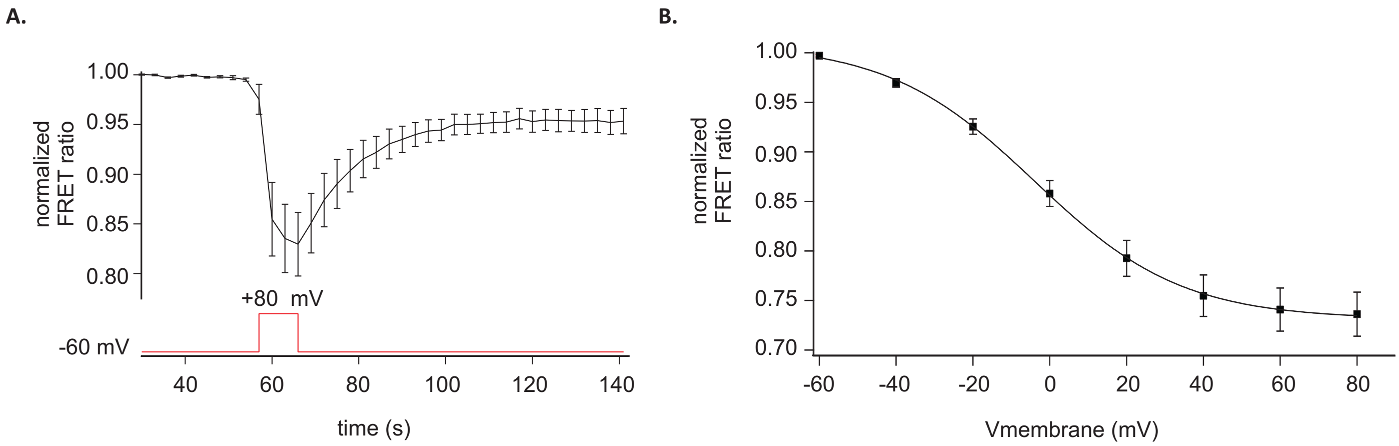

Supplementary Figure S3. Activation of $PTEN_{CiV}$ using K^+ dependent depolarization

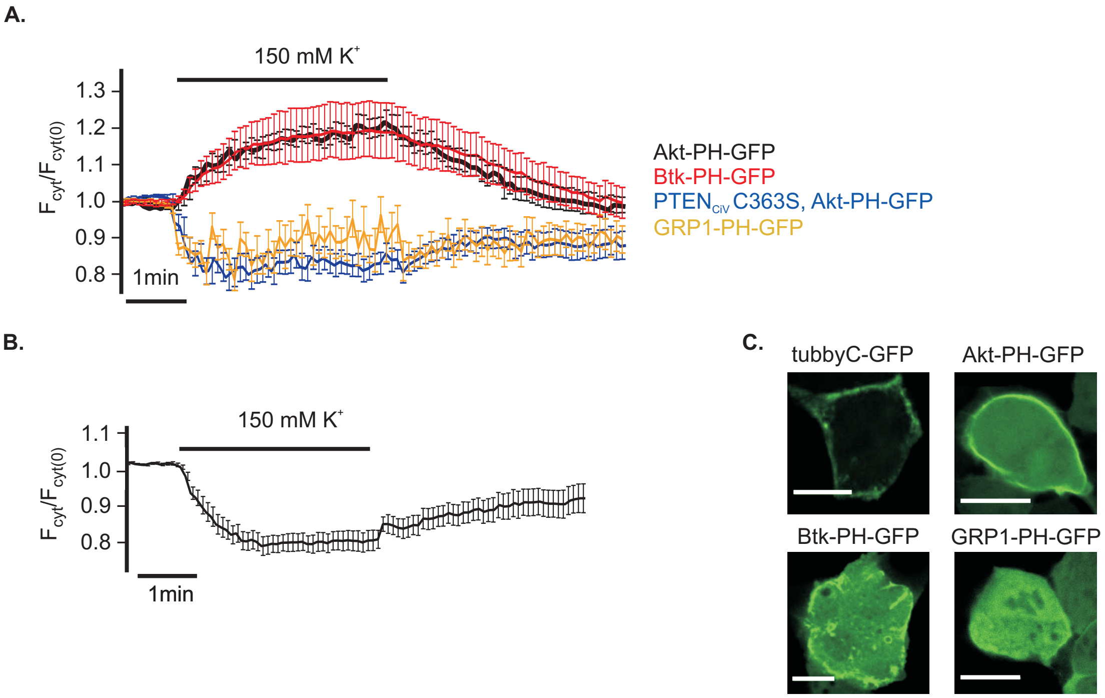

Supplementary Figure S4. Selection of a PI(3,4,5)P_3 marker for monitoring $PTEN_{CiV}$ activity

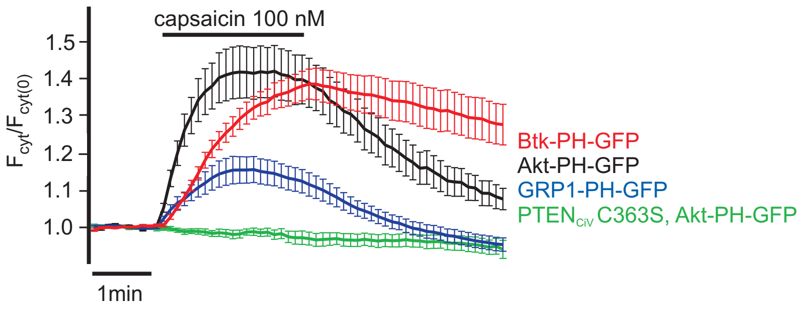

Supplementary Figure S5. PTEN$_{CiV}$ is inhibited by bisperoxovanadate compounds (data presented without re-normalization)

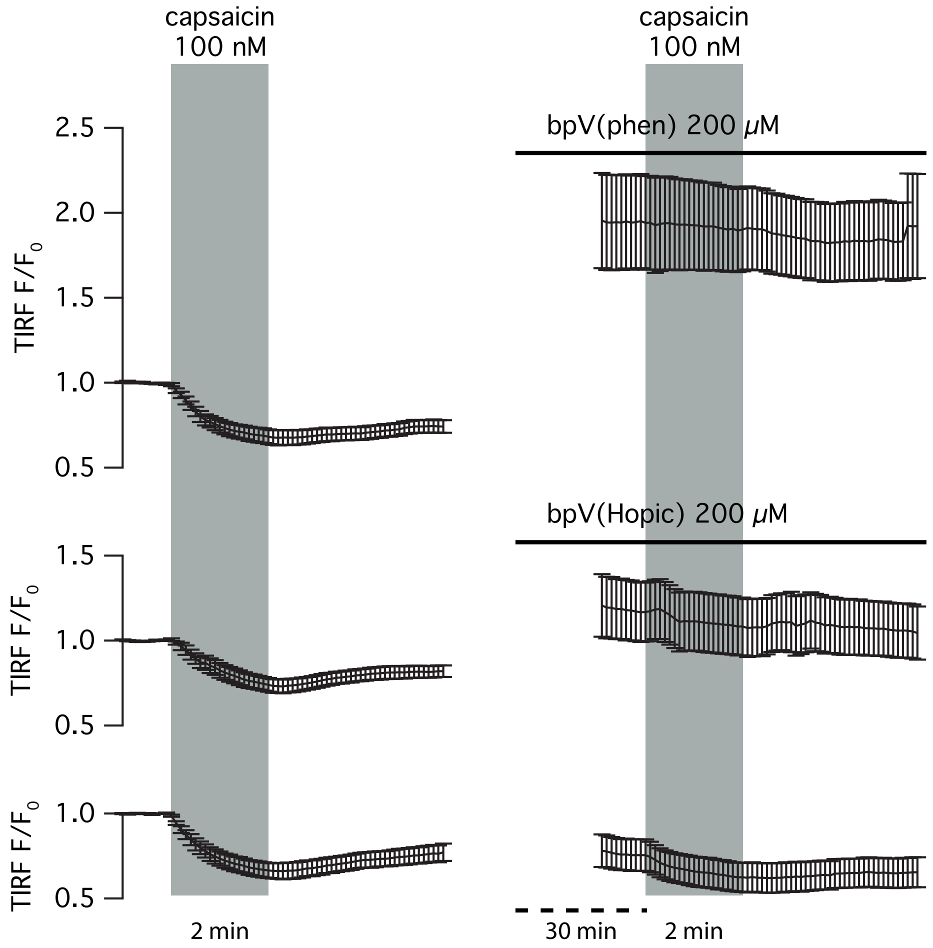

Supplementary Figure S6. bpV(phen) and bpV(HOpic) abolish $PTEN_{CiV}$ activity at micromolar concentrations when applied intracellularly

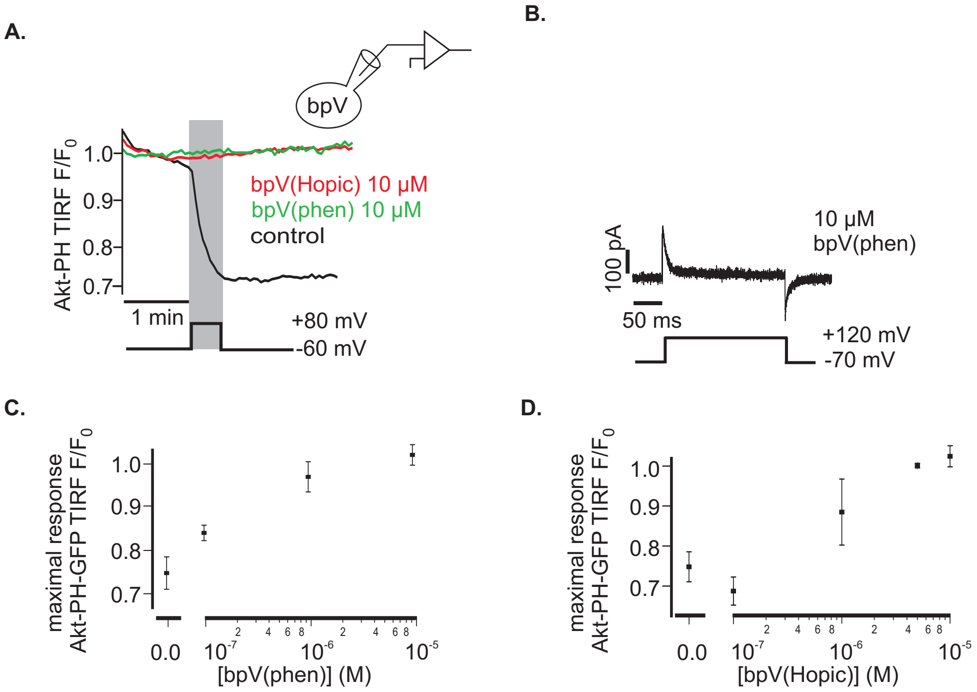

Supplement: Supplementary file 2 [file Image1.PDF]
